# Draft Genome Sequence of a New Homofermentative, Lactic Acid-Producing *Enterococcus faecalis* Isolate, CBRD01

**DOI:** 10.1128/genomeA.00147-14

**Published:** 2014-03-27

**Authors:** Lew P. Christopher, Vinayak Kapatral, Benjamin Vaisvil, Ginger Emel, Linda C. DeVeaux

**Affiliations:** a Center for Bioprocessing Research & Development and Civil & Environmental Engineering, South Dakota School of Mines & Technology, Rapid City, South Dakota, USA; b Igenbio, Inc., Chicago, Illinois, USA; c Department of Chemistry & Applied Biological Sciences, South Dakota School of Mines & Technology, Rapid City, South Dakota, USA

## Abstract

We report here the draft genome sequence of the novel homofermentative *Enterococcus faecalis* isolate CBRD01, which is capable of high lactic acid productivity and yields, with minimal nutritional requirements. The genome is 2.8 Mbp, with 37% G+C, and contains genes for two lactate dehydrogenase (LDH) enzymes found in related organisms.

## GENOME ANNOUNCEMENT

*Enterococcus faecalis*, a facultative Gram-positive anaerobe, is commonly found in the mammalian intestinal tract, human feces, dairy products, plants, and insects (1–4). It can survive in different environments at a wide range of temperatures, pH levels, and salinity (2, 5). *E. faecalis* secretes bacteriocins with bactericidal effects (6, 7), and it is used as food preservative and a probiotic to improve intestinal microbial balance (8–10). Because *E. faecalis* is also increasingly recognized as a multiresistant nosocomial pathogen, there is tremendous interest in *E. faecalis* genomes (11). There are currently >50 publicly available draft genomes (http://www.ncbi.nlm.nih.gov/genomeprj/20875), but few have been fully closed. Here, we report the draft genome sequence of a novel, nonfastidious, homolactic acid strain, *E. faecalis* CBRD01, isolated from municipal solid waste at the Rapid City Materials Recovery Facility. CBRD01 shows great promise in homofermentative lactic acid production at high yields, production rates, and purity on relatively simple fermentation medium, thereby minimizing production costs and pollution (L. P. Christopher, M. R. Subramanian, and S. Talluri, U.S. patent application 14/109,587). *E. faecalis* CBRD01 was deposited with the ATCC, under the patent deposition designation PTA-12846.

To understand the metabolic and pathogenic properties of *E. faecalis* CBRD01, the genome was sequenced and analyzed using the ERGO platform (12). Using Ion Torrent PGM technology (13), three sequencing runs were performed on a 316 Chip, yielding ~300 Mb of raw reads. *De novo*- and template-based assemblies were performed using *E. faecalis* OG1RF (accession no. CP002621). The genome of CBRD01 is 2,813,167 bp, with an average G+C content of 37%, assembled into 140 contigs. A total of 2,949 open reading frames (ORFs) (protein coding and RNA), including 40 tRNA species and three rRNA operons, were identified (14). Seventy-one percent of the ORFs have a functional annotation (12); approximately 20% utilize the alternate start codon TTG, compared to 8% in *E. faecalis* D32, V583, or OG1RF.

Like other *E. faecalis* strains, CBRD01 has two distinct ORFs for the key enzyme lactate dehydrogenase (LDH). ORF NHR02052 has a predicted length of 317 amino acids (aa), identical to orthologs in *E. faecalis* D32 and V583. ORF NHR01261 is predicted to be 403 aa, identical to *E. faecalis* OG1RF. This protein appears to utilize TTG rather than the standard ATG start codon. Unlike *E. faecalis* V583, there were no ORFs associated with xylose utilization; however, there are ORFs for glycerol, galactose, arabinose, sucrose, fructose, and maltose utilization.

We found one complete ORF (NHR01192) for pyruvate ferredoxin oxidoreductase (EC 1.2.7.1) and a second ORF that appears to have a frameshift (NHR01082 and NHR01932). We did not identify either pyruvate formate lyase or formate hydrogen lyase.

*E. faecalis* is resistant to ampicillin and vancomycin (15). CBRD01 also contains a factor for methicillin resistance (NHR01325), a multidrug resistance protein B, an efflux transporter regulator, and ORFs conferring resistance to β-lactams and aminoglycosides. We found only three transposase ORFs, similar to *E. faecalis* OG1RF (one transposase) but lower than the 33 in *E. faecalis* D32. Seventy-seven insertion sequence (IS) elements were identified, compared to 29 in *E. faecalis* OG1RF and 91 in *E. faecalis* D32, as well as at least three prophage sequences.

### Nucleotide sequence accession number.

The genome sequence of *E. faecalis* isolate CBRD01 has been deposited in GenBank under the accession no. AWYG01000000.
